# Mission in Sukusuku Cohort, Mie: A Study Focusing on the Characteristics of Participants and the Mental Health of the Mothers Raising Children

**DOI:** 10.2188/jea.JE20090167

**Published:** 2010-03-05

**Authors:** Noriko Yamakawa, Haruka Koike, Noriko Ohtani, Motoki Bonno, Shigeki Tanaka, Masaru Ido, Yoshihiro Komada, Masatoshi Kawai, Hatsumi Yamamoto

**Affiliations:** 1 Clinical Research Institute, Mie-chuo Medical Center, National Hospital Organization, Tsu, Japan; 2 Department of Neonatal Science, Institute of Molecular and Experimental Medicine, Mie University Graduate School of Medicine, Tsu, Japan; 3 Pediatrics and developmental Science, Institute of Molecular and Experimental Medicine, Mie University Graduate School of Medicine, Tsu, Japan; 4 Center for the study of child development, Institute for Education, Mukogawa Women’s University, Nishinomiya, Hyogo, Japan

**Keywords:** Japan Children’s Study, Sukusuku cohort, Mie, characteristics of participants, maternal mental health, childcare stress

## Abstract

**Background:**

We carried out Sukusuku cohort, Mie (SCM), a long term cohort study of child development and investigated the feasibility and validity of this study. Then we focused on the characteristics of the enrolled families and verified the representativeness of the participants in SCM.

**Methods:**

The characteristics of 185 families recruited from 3 hospitals were analyzed, and we verified the representativeness of these subjects. We also analyzed the factors that may influence the mental health of the mothers who are raising children.

**Results:**

There were no significant differences between the subjects from the 3 hospitals in terms of the age distribution, academic background, occupation, and annual income of the participating families. At 42 months, the average developmental quotients for postural and motor, cognitive and adaptive, and speech and social development in the 140 infants were 98.6, 100.6, and 99.9, respectively. The overall developmental quotient for infants was 100.3 ± 13.2; this score was within the standard range (55–132). The path-analysis model revealed that family function was an important factor influencing the mental health of mothers.

**Conclusions:**

The participant characteristics were thought to be generally representative, and we showed the validity and representativeness of the participants in this cohort study. The mental health analysis of mothers suggested that relieving mothers from child-rearing stress and maintaining family function were important for the maintenance and improvement of maternal mental health.

## INTRODUCTION

We had performed another study entitled “Mission in the Sukusuku Cohort Mie: Focusing on the feasibility and validity of methods for enrolling and retaining participants,”^1^ in which we had emphasized the importance of high enrollment and retention rates to maximize statistical reliability and minimize the bias attributed to dropouts in a longitudinal cohort study.^2^^,^^3^

Further, the study population in a cohort study that requires repeated observations should essentially remain representative of the general population.^2^ Therefore, we selected 3 medical institutes in different areas with low migration rates, so that the results could be compared with the national population. Several reports have suggested that impaired mental health of the mother due to child-rearing stress can delay child development.^4^^–^^7^ Therefore, we presumed that maternal mental health might play an important role in the mother’s participation in subsequent visits in a longitudinal study, and maternal mental health may be a crucial factor in obtaining responses to questionnaires, thereby securing the validity of child-rearing.

In this study, we analyzed the characteristics of the enrolled families and verified the representativeness of the participants. In addition, we performed a long-term study to investigate the factors that might influence the mental health of mothers raising children; for example, we determined whether the maternal stress occurring while rearing a 4-month-old child influences the stress occurring while raising the child at 9 months of age. This aspect of our study is of great interest in birth-cohort studies, and it would be of particular interest to neonatologists.

## METHODS

The recruitment details of the participants have been highlighted in our another paper.^1^

### Observations

Research rooms to observe child development were developed in Mie-chuo Medical Center, Tsu City, and Owase General Hospital, Owase City. The rooms contained an interview section, a waiting corner, a feeding room, and an observation booth. The children were observed by a pediatrician, clinical research coordinators (CRCs), and research personnel at 4, 9, 18, 30, and 42 months of age. From April 2005, the observations were performed in the observation booth (5.4 m × 5.4 m), which had a one-way mirror. The observation booth was equipped with 7 VTR cameras and a microphone, and a seat for the infant at the centre. The mother was seated on the floor in front of her infant. The cognitive and behavioral developments of the children were observed according to various protocols, which were developed and delivered by the development and brain science research group of JCS. The main observation procedures for the assessment of cognitive, physical, and mental developments and interaction with the mother or with strangers were also developed. Video recordings of the observations of each protocol for approximately 5–10 minutes were made. Digital video recordings were then sent to laboratories for further analysis and publication. Throughout the study, research personnel in SCM were trained and certified for each observation procedure to ensure reliable administration of all measures and to prevent variability in practices.

### Questionnaires

We designed questionnaires containing approximately 500 questions each for 4, 9, 18, 30, and 42 months of observation. These questionnaires were designed for JCS to obtain information about attitudes toward childcare, lifestyle, mental health, temperament, and developmental circumstances. These questionnaires were sent to the parents 2 weeks before each observation and were completed and submitted at the time of the visit. The results of the questionnaires were sent to the proponents of each research question for further analysis and publication.

### Analysis of the factors influencing maternal mental health

We studied the families of 126 infants born between August 2004 and November 2005 to analyze the factors influencing the mental health of their mothers. Data for the parents who provided consent at the 4- and 9-month observations were obtained through questionnaires. The questionnaire contained 10 questions, which aimed to determine the levels of child-rearing stress in mothers of 4-month-old infants. The questionnaires were used to determine whether the parents discussed child-rearing strategies, whether the mother was satisfied by the father’s involvement in raising the child, and the mothers’ self-evaluation of their involvement in raising the child. Further, we evaluated the family APGAR score and maternal mental health.^8^ The same questionnaire was used for assessing mothers of 4- or 9-month-old infants; however, mothers of 9-month-old infants were also evaluated using the family APGAR score and General Health Questionnaire (GHQ, Japanese version).^9^

### Statistical analysis

Statistical tests in group comparison of the characteristics of the participants were performed by the Student’s *t* test or chi-square test, and a *P* value < 0.05 was considered statistically significant. The partial regression coefficient was estimated in path analysis to examine the significance of factors that may influence maternal mental health. All analyses in this study were performed using SPSS Version 17 and AMOS 7.

## RESULTS

### Characteristics of the participants enrolled in the study in SCM

The characteristics of the participants were evaluated via questionnaires and are summarized in Table 1.

**Table 1.** Properties of the partcipating families

			all participants (*n* = 185)	participants of Mie-chuo Medical Center (*n* = 108)	participants of other clinic or Owase Hospital (*n* = 77)		
Fathers	age (year)		32.09 (± 5.19)				
							15–44 years male with jobs in Mie
	educational background	graduate of junior high school	8 (4.3)	6 (5.6)	2 (2.6)	N.S.	6.4%
		graduate of senior high school	60 (32.4)	37 (34.3)	23 (29.9)		47.9%
		graduate of university, college, junior ​ college, and vocational school	92 (49.7)	48 (44.4)	44 (57.1)		43.1%
		graduate of graduated school	14 (7.6)	9 (8.3)	5 (6.5)		2.6%
		no answer	11 (5.9)	8 (7.4)	3 (3.9)		
							15–44 years male with jobs in Mie
	occupational classification	Specialist and technical workers	68 (36.8)	34 (31.5)	34 (44.2)	N.S.	11.4%
		Administrative and managerial workers	8 (4.3)	3 (2.8)	5 (6.5)		1.3%
		Clerical workers	19 (10.3)	11 (10.2)	8 (10.4)		12.5%
		Sales workers	14 (7.6)	11 (10.2)	3 (3.9)		10.5%
		Service workers	13 (7.0)	10 (9.3)	3 (3.9)		5.5%
		Security workers	5 (2.7)	2 (1.9)	3 (3.9)		3.2%
		Agriculture, forestry and fishery workers	2 (1.1)	1 (0.9)	1 (1.3)		1.0%
		Transport and communication workers	7 (3.8)	3 (2.8)	4 (5.2)		5.0%
		Production process and related workers	22 (11.9)	14 (13.0)	8 (10.4)		46.2%
		other	11 (5.9)	7 (6.5)	4 (5.2)		3.3%
		Not engaged in work	1 (0.5)	1 (0.9)	0 (0.0)		0%
		no answer	15 (8.1)	11 (10.2)	4 (5.2)		0%

Mothers	age (year)		30.49 (± 4.42)				
							15–44 years female in Mie
	educational background	graduate of junior high school	6 (3.2)	6 (5.6)	0 (0.0)	N.S.	3.4%
		graduate of senior high school	46 (24.9)	28 (25.9)	18 (23.4)		43.3%
		graduate of university, college, junior ​ college, and vocational school	128 (69.2)	70 (64.8)	58 (75.3)		52.7%
		graduate of graduated school	2 (1.1)	1 (0.9)	1 (1.3)		0.6%
		no answer	3 (1.6)	3 (2.8)	0 (0.0)		
							15–44 years female in Mie
	occupational classification	Specialist and technical workers	45 (24.3)	30 (27.8)	15 (19.5)	N.S.	11.8%
		Administrative and managerial workers	0 (0.0)	0 (0.0)	0 (0.0)		0.0%
		Clerical workers	27 (14.6)	15 (13.9)	12 (15.6)		20.8%
		Sales workers	2 (1.1)	1 (0.9)	1 (1.3)		6.7%
		Service workers	3 (1.6)	2 (1.9)	1 (1.3)		9.4%
		Security workers	1 (0.5)	0 (0.0)	1 (1.3)		0.2%
		Agriculture, forestry and fishery workers	1 (0.5)	0 (0.0)	1 (1.3)		0.2%
		Transport and communication workers	0 (0.0)	0 (0.0)	0 (0.0)		0.2%
		Production process and related workers	0 (0.0)	0 (0.0)	0 (0.0)		11.2%
		other	5 (2.7)	3 (2.8)	2 (2.6)		2.1%
		Not engaged in work	88 (47.6)	50 (46.8)	38 (49.4)		37.3%
			13 (7.0)	7 (6.5)	6 (7.8)		

	household annual income	less than 2 million yen	3 (1.6)	2 (1.9)	1 (1.3)	N.S.	average of Japanese household annual income
		2–4 million yen	46 (24.9)	20 (18.5)	26 (33.8)		
		4–6 million yen	61 (33.3)	40 (37.0)	21 (27.3)		5.50 million yen
		6–8 million yen	26 (14.1)	17 (15.7)	9 (11.7)		
		8–10 million yen	15 (8.1)	8 (7.4)	7 (9.1)		
		above 10 million yen	11 (5.9)	5 (4.6)	6 (7.8)		
		no answer	23 (12.4)	16 (14.8)	7 (9.1)		

Babies	order of birth	first	94 (50.8)	56 (51.9)	38 (49.4)		
		second	68 (36.8)	42 (38.9)	26 (33.8)		
		third	23 (12.4)	10 (9.3)	13 (16.9)		
		fourth and more	0 (0.0)	0 (0.0)	0 (0.0)		

	gestational age (weeks)		39.0	38.8	39.2		

	boys:girls		90:95	49:59	41:36		
							Japanese babies born in 2000
	weight at birth (boys) (kg)		3.04 (± 0.39)	2.92 (± 0.39)	3.19 (± 0.33)		3.00 (2.76)
	weight at birth (girls) (Kg)		2.93 (± 0.36)	2.94 (± 0.38)	2.92 (± 0.32)		2.95 (2.72)
	height at birth (boys) (cm)		49.8 (± 1.9)	49.8 (± 2.1)	49.8 (± 1.6)		49.0 (47.7)
	height at birth (girls) (cm)		49.0 (± 1.8)	49.6 (± 1.8)	48.1 (± 1.2)		48.5 (47.3)
	head circumference ​ at birth (boys) (cm)		33.5 (± 1.3)	33.2 (± 1.2)	33.9 (± 1.9)		33.5 (32.7)
	head circumference ​ at birth (girls) (cm)		32.9 (± 1.3)	32.9 (± 1.3)	33.0 (± 1.2)		33.0 (32.2)

Values are numbers (percentages), means (± SDs), and 50 percentile (25 percentile).

#### Perinatal conditions of the participating infants

Among the 467 families that were deemed eligible to participate in this study, 199 (42.6%) provided consent; however, 14 of these families withdrew consent, and finally, 185 families (39.6%) participated at 4 months of observation.

A total of 140 (75.7%) infants were born in hospitals, and 45 (24.3%) infants were born in a maternity clinic. The gestational age ranged from 36 to 42 weeks (mean: 39.0 weeks), and the body weight at birth ranged from 2076 to 4156 g (mean: 2987.0 g). Among these infants, 5 (2.7%) were born at 36 weeks of gestational age, and the body weight at birth of 15 (8.1%) infants was less than 2500 g. Five (2.7%) infants had mild birth asphyxia. Twenty-nine (15.7%) infants were delivered by cesarean section. The duration of hospitalization ranged from 4 to 16 days.

#### Birth order

Among the 185 infants assessed in this study, 94 (50.8%) were first-born, 68 (36.8%) were second-born, and 23 (12.4%) were third-born; there were no later-born infants. These results were in agreement with the vital statics from the Ministry of Health, Labor and Welfare, Japan,^10^ according to which the rates of first-, second-, and later-born infants were 48.2%, 37.6%, and 14.2%, respectively.

#### Parental age

The paternal age ranged from 20 to 58 years (mean ± SD: 32.09 ± 5.19 years), and 41.1%, 21.6%, and 17.3% of the fathers belonged to the age brackets of 30–34 years, 35–39 years, and 25–29 years, respectively. The maternal age ranged from 20 to 41 years (mean ± SD: 30.49 ± 4.42), and 47.0%, 24.9%, and 12.4% belonged to the age ranges of 30–34 years, 25–29 years, and 35–39 years, respectively.

#### Academic background of the parents

A survey revealed that the achieved level of education in 4.3%, 32.4%, 49.7%, and 7.6% of the fathers was junior high school, high school, university, and graduate school, respectively. Similarly, the achieved level of education in 3.2%, 24.9%, 69.2%, and 1.1% of the mothers in this study was junior high school, high school, university, and graduate school, respectively.

#### Occupation

The survey revealed that 36.8% of the fathers were professionals and technicians; 11.9%, factory workers; and 10.3%, office workers. There was only 1 unemployed father among the participants. Further, the survey revealed that 47.6%, 24.3%, and 14.6% of the mothers were unemployed and doing housework, professionals and technicians, and office workers, respectively.

#### Annual income

The annual family income of 1.6%, 24.9%, 33.0%, 14.1%, 8.1%, and 5.9% of the participants was <2, 2–4, 4–6, 6–8, 8–10, and >10 million yen, respectively.

There was no significant difference in the age distribution, academic background, occupation, and annual income of the participating families recruited from Mie-chuo Medical Center and the other hospitals.

#### Twins

Three sets of twins, who were recruited from Mie-chuo Medical Center, were enrolled in this study.

#### Child development

The average developmental quotients of the 140 participants who were evaluated using the Kyoto Scale of Psychological Development at 42 months of age were 98.6 ± 11.4 (range: 57–110) in the fields of postural and motor development, 100.6 ± 13.8 (range: 57–134) in the fields of cognitive and adaptive development, and 99.9 ± 15.5 (range: 54–135) in the fields of speech and social development. The overall developmental quotient of the participants was 100.3 ± 13.2 (range: 55–132), which was estimated to be within the standard range. However, 1 infant was diagnosed with pervasive developmental disorder at 18 months of age. Three infants were suggested to have mental retardation, attention deficit/hyperactivity disorder, or developmental motor-coordination disorder.

### Factors that may influence maternal mental health

**Figure 1.**
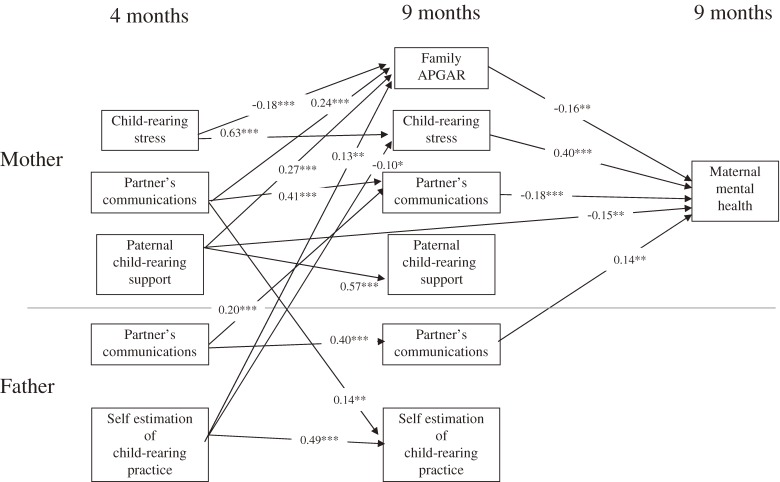
Final model of the factors influencing maternal mental health

For the path-analysis model, we hypothesized that the factors assessed when the infant is 4 months of age may influence the mental health of the mother when the infant is 9 months of age (Figure 1). The partial regression coefficients were estimated to examine the significance of these factors. A final model was defined after excluding the insignificant path. The findings of this final model showed that family function was an important factor influencing maternal mental health. Improved family function resulted in better mental health with lower child-rearing stress. These analyses confirmed the hypothesis that child-rearing stress associated with the rearing of a 9-month-old infant was influenced by the child-rearing stress occurring when the infant was 4 months of age.

## DISCUSSION

When participants are recruited at hospitals, their characteristics are strongly affected by the type of hospital. Since Mie-chuo Medical Center is a public hospital and a perinatal medical center for mothers and children, some of the characteristics were biased, eg, high-risk pregnancies, earnestly desired babies, and low family income. Therefore, to avoid these biases, we recruited participants from several medical institutes. Performing a longitudinal cohort study over one or more geographically defined areas is more advantageous than attempting to enroll a national sample,^11^ but the chosen areas should have low outward-migration rates.

We analyzed the age of parents and the academic background, occupation, and annual family income of the participants. There were no differences between the values and distributions of all these characteristics and the data from the national survey; further, there were no significant differences between the values obtained from the 3 hospitals. The educational background and professional jobs of the parents in our study were higher than the corresponding data from the national survey; however, this difference was not confirmed statistically. These results confirmed the representativeness of the participants and the validity of the recruitment procedures and retention strategies of our study.

Our findings revealed that ensuring relief from child-rearing stress and maintaining family function were important for the maintenance and improvement of the mental health of the mothers. Moreover, this study confirmed the hypothesis that the child-rearing stress occurring while the infant was 9 months old was influenced by the factors that occurred when the child was 4 months of age. Furthermore, this result indicated the continuity of maternal stress, which must be reduced from an early age to maintain maternal mental health. Mothers who brought up 2 or more children were less prone to childcare stress than mothers who brought up 1 child alone. However, these mothers appeared to receive inappropriate family support for childcare. The type of support required by the mother depends on the age of the child.^12^ Child-rearing support provided by the husband was considered to be an effective factor for maintaining the mental health of the mother, but maternal recognition of the husband’s support was more effective in reducing maternal mental stress. The findings of this study suggested that good maternal mental health was related to good communication between the parents and good child-rearing practices. Child rearing is important for infantile development, and well-conditioned early development plays a key role in the later development of the children.^13^^,^^14^ Psychological stress of mothers must be detected and analyzed at an early stage, because maternal stress affects the cognitive development of the children.

## CONCLUSION

The participant characteristics were thought to be generally representative of the national population, and we showed the reliability and the representativeness of the participants in this cohort study. The final path-analysis model revealed that family function was an important factor in the mental health of mothers and suggested that relieving the mothers from child-rearing stress and maintaining family function were important factors in the maintenance and improvement of maternal mental health.
